# Supplementary material for: Machine-learning approach expands the repertoire of anti-CRISPR protein families
Source: Nat Commun. 2020 Jul 29;11:3784. doi: 10.1038/s41467-020-17652-0 (PMC7391736; doi:10.1038/s41467-020-17652-0)
Supplement: Supplementary file 1 — Supplementary Information [file 41467_2020_17652_MOESM1_ESM.pdf]

Supplementary Information for

Machine-learning approach expands the repertoire of anti-CRISPR protein families

Gussow et al.

Supplementary Figures

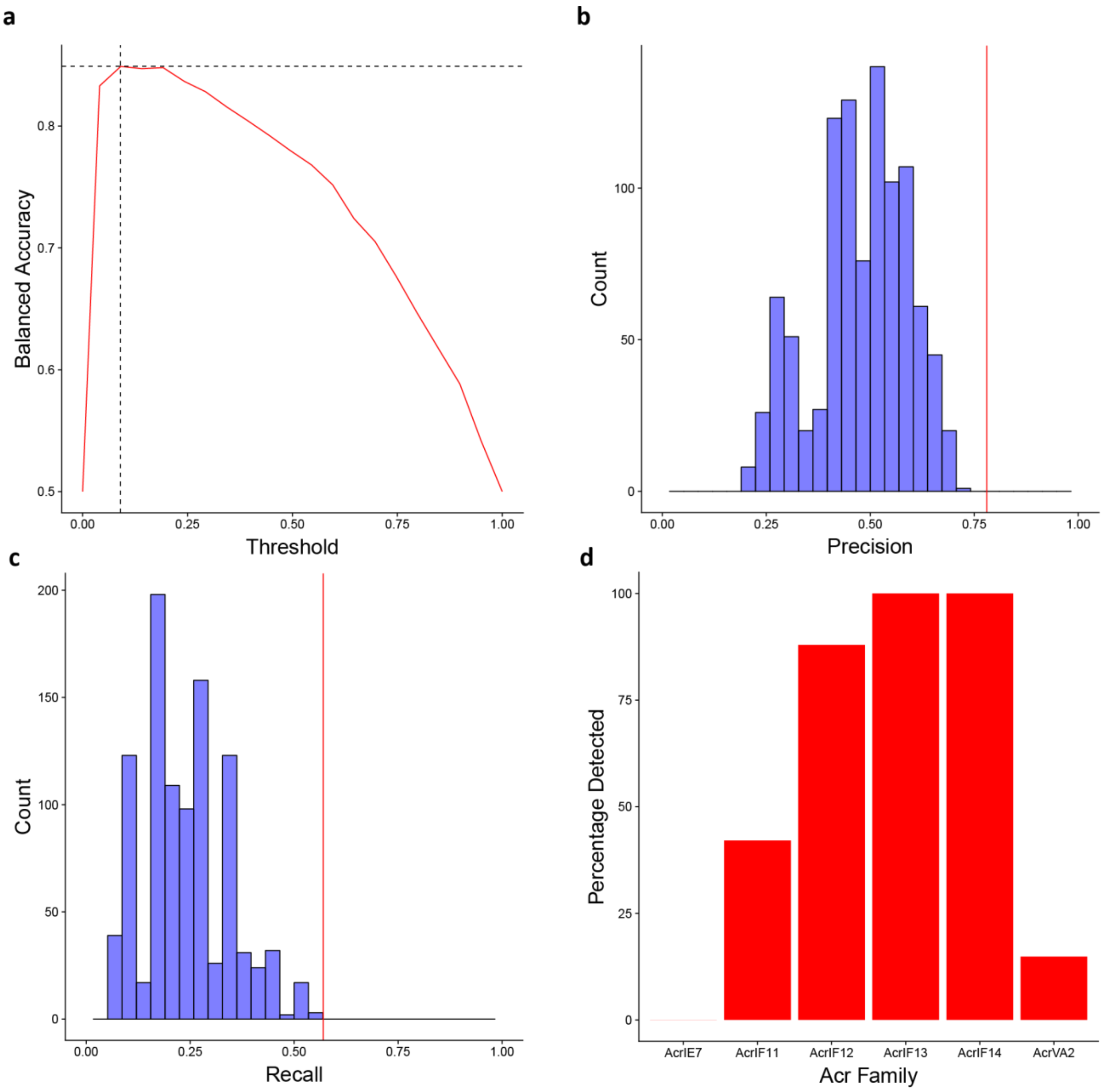

Supplementary Figure 1. Performance of binary classifier**.**

(a) Plot of the balanced accuracy across thresholds. The vertical dashed line indicates the threshold that achieves the highest balanced accuracy in the test set (0.09) while the horizontal dashed line indicates the highest balanced accuracy achieved (0.849). The lowest balanced accuracy (0.5, equivalent to a random classifier) occurs at thresholds of 0 and 1 where all samples are classified as the same class.

(b) and **(c)** Histograms of 1000 precisions and recalls calculated using permuted model classifications, representing the null precision and recall distributions. The red lines denote the true precision and true recall. The null precision is centered at 0.5, while the null recall is centered at 0.25.

(d) Bar plot of percentages of Acr families detected from the test data, using the binary classifier.

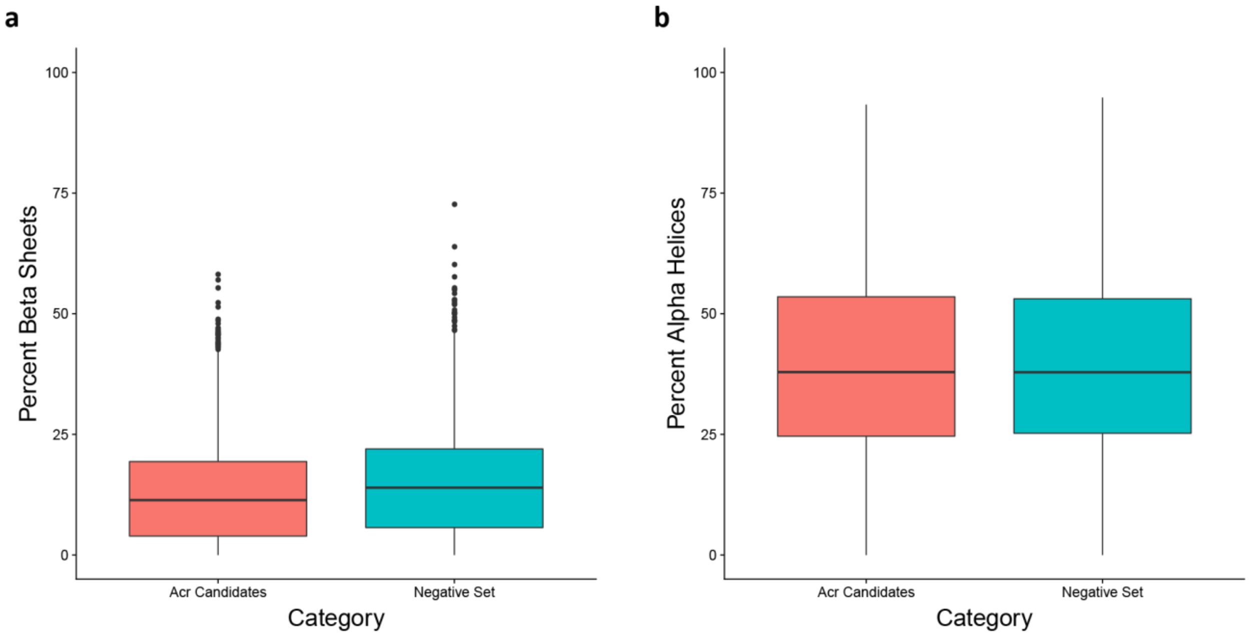

Supplementary Figure 2. Beta sheets and alpha helices.
(a) and **(b)** Boxplots of the percentage of amino acids contributing to beta sheets and alpha helices, comparing between Acr candidates (n=2,500 protein sequences) and the negative set (n=2,547 protein sequences). The horizontal black line is the median value. The lower and upper hinges correspond to the first and third quartiles, while the whiskers extend from the hinges to the largest and lowest values, no further than 1.5 times the distance between the first and third quartiles. Data beyond the end of the whiskers are plotted individually. Source data are provided as a Source Data file.

Supplementary Tables

Acr Family	Sample Sequence	Number of Members in Our Database	Used in Test Set
acric1	MNNLKKTAITHDGVFAYKNTETVIGSVGRNDIVMAIDATHGEFNDKNFIIYADTNGNPIYLGYAYLDDNNDAHIDLAVGACNEDDDFDEKEIHEMIAEQMELAKRYQELGDTVHGTTRLAFDDDGYMTVRLDQQAYPDYRPENDDKHIMWRALALTATGKELEVFWLVEDYEDEEVNSWDFDIADDWREL	0	FALSE
acrid1	MNYKELEKMLDVIFENSEIKEIDLFFDPEVEISKQEFEDLVKNADPLQKVVGDNYITETFEWWEFENQYLEFELDYYVKDEKIFVLEMHFWRKIRKLE	15	FALSE
acrie1	MEKKLSDAQVALVAAWRKYPDLRESLEEAASILSLIVFQAETLSDQANELANYIRRQGLEEAEGACRNIDIMRAKWVEVCGEVNQHGIRVYGDAIDRDVD	180	FALSE
acrie2	MNTYLIDPRKNNDNSGERFTVDAVDITAAAKSAAQQILGEEFEGLVYRETGESNGSGMFQAYHHLHGTNRTETTVGYPFHVMEL	34	FALSE
acrie3	MKITNDTTTYEVAELMGSEADELDGRIMMGLLSRECVVDTDDLSEDQWLALIDESQKVRREQFESDEA	377	FALSE
acrie4	MSTQYTYEQIAEDFRLWGEYMDPNAEMTEEEFQALSTEEKVAMQVEAFGAEA	90	FALSE
acrie5	MSNDRNGIINQIIDYTGTDRDHAERIYEELRADDRIYFDDSVGLDRQGLLIREDVDLMAVAAEIE	0	FALSE
acrie6	MNNDTEVLEQQIKAFELLADELKDRLPTLEILSPMYTAVMVTYDLIGKQLASRRAELIEILEEQYPGHAADLSIKNLCP	0	FALSE
acrie7	MIGSEKQVNWAKSIIEKEVEAWEAIGVDVREVAAFLRSISDARVIIDNRNLIHFQSSGISYSLESSPLNSPIFLRRFSACSVGFEEIPTALQRIRSVYTAKLLEDE	1	TRUE
acrif1	MKFIKYLSTAHLNYMNIAVYENGSKIKARVENVVNGKSVGARDFDSTEQLESWFYGLPGSGLGRIENAMNEISRRENP	61	FALSE
acrif10	MTTFRIENVRIETINDFDMVKFDLVTDLGRVELAEHVNYDSEGDFKSVEYTDSNIRYNMVDELCSVFDLTDKPSLMPAIDYVTFAEIIEAVEEMLEA	2	FALSE
acrif11	MSMELFHGSYEEISEIRDSGVFGGLFGAHEKETALSHGETLHRIISPLPLTDYALNYEIESAWEVALDVAGGDENVAEAIMAKACESDSNDGWELQRLRGVLAVRLGYTSVEMEDEHGTTWLCLPGCTVEKI	136	TRUE
acrif12	MAYEKTWHRDYAAESLKRAETSRWTQDANLEWTQLALECAQVVHLARQVGEELGNEKIIGIADTVLSTIEAHSQATYRRPCYKRITTAQTHLLAVTLLERFGSARRVANAVWQLTDDEIDQAKA	262	TRUE
acrif13	MKLLNIKINEFAVTANTEAGDELYLQLPHTPDSQHSINHEPLDDDDFVKEVQEICDEYFGKGDRTLARLSYAGGQAYDSYTEEDGVYTTNTGDQFVEHSYADYYNVEVYCKADLV	2	TRUE
acrif14	MKKIEMIEISQNRQNLTAFLHISEIKAINAKLADGVDVDKKSFDEICSIVLEQYQAKQISNKQASEIFETLAKANKSFKIEKFRCSHGYNEIYKYSPDHEAYLFYCKGGQGQLNKLIAENGRFM	2	TRUE
acrif2	MIAQQHKDTVAACEAAEAIAIAKDQVWDGEGYTKYTFDDNSVLIQSGTTQYAMDADDADSIKGYADWLDDEARSAEASEIERLLESVEEE	43	FALSE
acrif3	MSSTISDRIISRSVIEAARFIQSWEDADPDNLTESQVLAASSFAARLHEGLQATVLQRLVDESNRDEYREFQAWEEALLNADGRVTSNPFADWGWWYRIANVMLATASQNVGVAWGSHVHGRLMAIFQDRFQQHYEDEEC	429	FALSE
acrif4	MMTISKTDIDCYLQTYVVIDPVSNGWQWGIDENGVGGALHHGRVEMVEGENGYFGLRGATHPTEKEAMAAALGYLWKCRQDLVAIARNDAIEAEKYRAKA	356	FALSE

acrif5	MSRPTVVTVTETPRNPGSYEVNVERDGKMVVGRARAGSDPGAAAAKAMQMAMEWGSPNYVILGSNKVLAFIPEQLRVKM	110	FALSE
acrif6	MKVPAFFAANILTIEQIIEAINNDGSAMTSAPEIAGYYAWDAATDALESENDLEQLTEDDFVAHLEVLEERGAKIDRDAAIAVALQFQAAAVNDLHSGDE	35	FALSE
acrif7	MSHASHNGEAPKRIEAMTTFTSIVTTNPDFGGFEFYVEAGQQFDDSAYEEAYGVSVPSAVVEEMNAKAAQLKDGEWLNVSHEA	47	FALSE
acrif8	MARIAPNEDSTMSTAYIIFNSSVAAVVDTEIANGANVTFSTVTVKEEINANRDFNLVNAQNGKISRAKRWGNEASKCEYFGREINPTEFFIK	4	FALSE
acrif9	MKAAYIIKEVQNINSEREGTQIEATSLSQAKRIASKEQCFHGTVMRIETVNGLWLAYKEDGKRWVDCQ	26	FALSE
acriia1	MTIKLLDEFLKKHDLTRYQLSKLTGISQNTLKDQNEKPLNKYTVSILRSLSLISGLSVSDVLFELEDIEKNSDDLAGFKHLLDKYKLSFPAQEFELYCLIKEFESANIEVLPFTFNRFENEEHVNIKKDVCKALENAITVLKEKKNELL	205	FALSE
acriia2	MTLTRAQKKYAEAMHEFINMVDDFEESTPDFAKEVLHDSDYVVITKNEKYAVALCSLSTDECEYDTNLYLDEKLVDYSTVDVNGVTYYINIVETNDIDDLEIATDEDEMKSGNQEIILKSELK	146	FALSE
acriia3	MFNKAEIMKQAWNWFNDSNIWLSDIEWVSYTDKEKSFSVCLKAAWSKAKEEVEESKKESKHIAKSEELKAWNWAERKLGLHFNISDDEKFTSVKDETKINFGLSVWACAMKAVKLHNDLFPQTAA	101	FALSE
acriia4	MNINDLIREIKNKDYTVKLSGTDSNSITQLIIRVNNDGNEYVISESENESIVEKFISAFKNGWNQEYEDEEEFYNDMQTITLKSELN	59	FALSE
acriia5	MAYGKSRYNSYRKRSFNRSNKQRREYAQEMDRLEKAFENLDGWYLSSMKDSAYKDFGKYEIRLSNHSADNKYHDLENGRLIVNIKASKLNFVDIIENKLDKIIEKIDKLDLDKYRFINATNLEHDIKCYYKGFKTKKEVI	11	FALSE
acriia6	MKINDDIKELILEYMSRYFKFENDFYKLPGIKFTDANWQKFKNGGTDIEKMGAARVNAMLDCLFDDFELAMIGKAQTNYYNDNSLKMNMPFYTYYDMFKKQQLLKWLKNNRDDVIGGTGRMYTASGNYIANAYLEVALESSSLGSGSYMLQMRFKDYSKGQEPIPSGRQNRLEWIENNLENIR	23	FALSE
acriic1	MANKTYKIGKNAGYDGCGLCLAAISENEAIKVKYLRDICPDYDGDDKAEDWLRWGTDSRVKAAALEMEQYAYTSVGMASCWEFVEL	11	FALSE
acriic2	MSKNNIFNKYPTIIHGEARGENDEFVVHTRYPRFLARKSFDDNFTGEMPAKPVNGELGQIGEPRRLAYDSRLGLWLSDFIMLDNNKPKNMEDWLGQLKAACDRIAADDLMLNEDAADLEGWDD	400	FALSE
acriic3	MFKRAIIFTSFNGFEKVSRTEKRRLAKIINARVSIIDEYLRAKDTNASLDGQYRAFLFNDESPAMTEFLAKLKAFAESCTGISIDAWEIEESEYVRLPVERRDFLAAANGKEIFKI	7	FALSE
acriic4	MKITSSNFATIATSENFAKLSVLPKNHREPIKGLFKSAVEQFSSARDFFKNENYSKELAEKFNKEAVNEAVEKLQKAIDLAEKQGIQF	1	FALSE
acriic5	MNNSIKFHVSYDGTARALFNTKEQAEKYCLVEEINDEMNGYKRKSWEEKLREENCASVQDWVEKNYTSSYSDLFNICEIEVSSAGQLVKIDNTEVDDFVENCYGFTLEDDLEEFNKAKQYLQKFYAECEN	2	FALSE
acrva1	MYEAKERYAKKKMQENTKIDTLTDEQHDALAQLCAFRHKFHSNKDSLFLSESAFSGEFSFEMQSDENSKLREVGLPTIEWSFYDNSHIPDDSFREWFNFANYSELSETIQEQGLELDLDDDETYELVYDELYTEAMGEYEELNQDIEKYLRRIDEEHGTQYCPTGFARLR	0	FALSE
acrva2	MHHTIARMNAFNKAFANAKDCYKKMQAWHLLNKPKHAFFPMQNTPALDNGLAALYELRGGKEDAHILSILSRLYLYGAWRNTLGIYQLDEEIIKDCKELPDDTPTSIFLNLPDWCVYVDISSAQIATFDDGVAKHIKGFWAIYDIVEMNGINHDVL	476	TRUE

	DFVVDTDTDDNVYVPQPFILSSGQSVAEVLDYGASLFDDDTSNTLIKGLLPYLLWLCVAEPDITYKGLPVSREELTRPKHSINKKTGAFVTPSEPFIYQIGERLGSEVRRYQSIIDGEQKRNRPHTKRPHIRRGHWHGYWQGTGQAKEFRVRWQPAVFVNSGRVSS		
acrva3	MVGKSKIDWQSIDWTKTNAQIAQECGRAYNTVCKMRGKLGKSHQGAKSPRKDKGISRPQPHLNRLEYQALATAKAKASPKAGRFETNTKAKTWTLKSPDNKTYTFTNLMHFVRTNPHLFDPDDVVWRTKSNGVEWCRASSGLALLAKRKKAPLSWKGWRLISLTKDNK	0	FALSE
acrva4	MYEIKLNDTLIHQTDDRVNAFVAYRYLLRRGDLPKCENIARMYYDGKVIKTDVIDHDSVHSDEQAKVSNNDIIKMAISELGVNNFKSLIKKQGYPFSNGHINSWFTDDPVKSKTMHNDEMYLVVQALIRACIIKEIDLYTEQLYNIIKSLPYDKRPNVVYSDQPLDPNNLDLSEPELWAEQVGECMRYAHNDQPCFYIGSTKRELRVNYIVPVIGVRDEIERVMTLEEVRNLHK	0	FALSE
acrva5	MKIELSGGYICYSIEEDEVTIDMVEVTTKRQGIGSQLIDMVKDVAREVGLPIGLYAYPQDDSISQEDLIEFYFSNDFEYDPDDVDGRLMRWS	0	FALSE

Supplementary Table 1. Known Acr families.

Family	Count	Percent predicted
AcrIE7	1	0
AcrIF11	136	42.091
AcrIF12	262	87.931
AcrIF13	2	100
AcrIF14	2	100
AcrVA2	476	14.841

Supplementary Table 2. Test set prediction percentages per Acr family.

name	Acr Families	Filter B	Filter C	Filter D
cluster_269	acrie3	TRUE	TRUE	TRUE
cluster_77558	acrif6	FALSE	FALSE	FALSE
cluster_138714	acriic1	FALSE	FALSE	FALSE
cluster_104567	acriic1	FALSE	FALSE	FALSE
cluster_210338	acrif8	TRUE	TRUE	TRUE
cluster_211799	acriic5	FALSE	FALSE	FALSE
cluster_22268	acrif3	FALSE	FALSE	FALSE
cluster_21454	acriia4	FALSE	FALSE	FALSE
cluster_14904	acriia4	FALSE	FALSE	FALSE
cluster_8039	acriic2	FALSE	FALSE	FALSE
cluster_7882	acrif1	TRUE	TRUE	TRUE
cluster_7662	acrie2	TRUE	FALSE	FALSE
cluster_6117	acrif6	FALSE	FALSE	FALSE
cluster_6088	acrie4	TRUE	TRUE	TRUE
cluster_5016	acrie1	TRUE	TRUE	TRUE
cluster_4463	acrif5	TRUE	TRUE	TRUE
cluster_2776	acriic2	TRUE	FALSE	FALSE
cluster_2194	acriia3	TRUE	TRUE	TRUE

cluster_1319	acrif3	TRUE	TRUE	TRUE
cluster_4721	acrif2	TRUE	TRUE	TRUE
cluster_33858	acriic2	TRUE	TRUE	TRUE
cluster_72142	acrif9	FALSE	FALSE	FALSE
cluster_66865	acrif8	FALSE	FALSE	FALSE
cluster_45387	acriic1	TRUE	TRUE	TRUE
cluster_42694	acriia2	TRUE	TRUE	TRUE
cluster_34111	acriic3	TRUE	TRUE	TRUE
cluster_1021	acriia2	TRUE	TRUE	TRUE
cluster_4724	acriia4	TRUE	TRUE	TRUE
cluster_16728	acrif11	FALSE	FALSE	FALSE
cluster_69102	acrif11	FALSE	FALSE	FALSE
cluster_3855	acrif12	TRUE	TRUE	FALSE
cluster_23694	acrif11	TRUE	TRUE	TRUE
cluster_39428	acrif11	TRUE	TRUE	TRUE

Supplementary Table 3. Heuristic filtering of Acrs in candidate set.

name	percentage
CAS-I-E	27.9
CAS-I-C	23.76
CAS-I-B	22.22
CAS-II-C	10.61
CAS-I-F	7.98
CAS-II-A	7.16
CAS-III-D	7.04
CAS-III-A	7.03
CAS-III-B	7.01
CAS-I-U	3.48
CAS-I-D	1.44
CAS-IV-A	1.41
CAS-III-C	1.15
CAS-VI-A	1.08
CAS-I-A	1.04
CAS-V-A	1.04
CAS-V-B	0.59
CAS-VI-B	0.53
CAS-VI-C	0.2
CAS-II-B	0.15

Supplementary Table 4. CRISPR-Cas subtypes present in genomes containing predicted Acrs.

	Type I-C	Type I-E	Type I-F
cluster_23907	AcrIC9		
cluster_11640	AcrIC10		
cluster_16134	*		
cluster_40699	AcrIC4	NA	NA
cluster_23719			
cluster_25827	AcrIC3	NA	NA
cluster_29540			
cluster_26949			
cluster_1797			
cluster_6290			
cluster_129519			
cluster_230863			
cluster_65661			
cluster_183357			
cluster_2684			
cluster_105837			
cluster_29205			
cluster_216046			
cluster_25901	†		
cluster_5186			
cluster_17920	†		
cluster_13736			
cluster_436			
cluster_31854			
cluster_135321			
cluster_32828	*		
cluster_11279			
cluster_152393			
cluster_49593			
cluster_29145			
cluster_47132			

Supplementary Table 5. Candidates selected for testing. Yellow indicates positive results from this study, green indicates positive results from Leon et al[1], red indicates negative results, black indicates failure to clone, an asterisk indicates failed transformation, and a cross indicates failed plaque assay.

Supplementary References

1. Leon, L.M., Park, A.E., Borges, A.L., Zhang, J. & Bondy-Denomy, J. Mobile element warfare via crispr and anti-crispr in pseudomonas aeruginosa. *Preprint at: https://www.biorxiv.org/content/10.1101/2020.06.15.151498v1* (2020).
