# Supplementary material for: Machine-learning approach expands the repertoire of anti-CRISPR protein families
Source: Nat Commun. 2020 Jul 29;11:3784. doi: 10.1038/s41467-020-17652-0 (PMC7391736; doi:10.1038/s41467-020-17652-0)
Supplement: Supplementary file 3 — Description of Additional Supplementary Files [file 41467_2020_17652_MOESM3_ESM.docx]

**Description of Additional Supplementary Files**

**File Name: Supplementary Data 1**

**Description:** Positive and negative sets of proteins used in training and validation.

**File Name: Supplementary Data 2**

**Description:** Predicted protein clusters, available at <ftp://ftp.ncbi.nih.gov/pub/wolf/_suppl/ACR20/supplementary_file_2.txt>.

**File Name: Supplementary Data 3**

**Description:** Top 30 candidates and their features.

**File Name: Supplementary Data 4**

**Description:** List of self-targeting assemblies.

**File Name: Supplementary Data 5**

**Description:** Fasta of Acr-related sequences.
