# Supplementary material for: Machine-learning approach expands the repertoire of anti-CRISPR protein families
Source: Nat Commun. 2020 Jul 29;11:3784. doi: 10.1038/s41467-020-17652-0 (PMC7391736; doi:10.1038/s41467-020-17652-0)
Supplement: Supplementary file 9 — Reporting Summary [file 41467_2020_17652_MOESM9_ESM.pdf]

nature research

Corresponding author(s): Eugene V. Koonin

Last updated by author(s): Jul 1, 2020

Reporting Summary

Nature Research wishes to improve the reproducibility of the work that we publish. This form provides structure for consistency and transparency in reporting. For further information on Nature Research policies, see our Editorial Policies and the Editorial Policy Checklist.

Statistics

For all statistical analyses, confirm that the following items are present in the figure legend, table legend, main text, or Methods section.

n/a	Confirmed	
☐	☒	The exact sample size (*n*) for each experimental group/condition, given as a discrete number and unit of measurement
☐	☒	A statement on whether measurements were taken from distinct samples or whether the same sample was measured repeatedly
☐	☒	The statistical test(s) used AND whether they are one- or two-sided<br>*Only common tests should be described solely by name; describe more complex techniques in the Methods section.*
☐	☒	A description of all covariates tested
☒	☐	A description of any assumptions or corrections, such as tests of normality and adjustment for multiple comparisons
☐	☒	A full description of the statistical parameters including central tendency (e.g. means) or other basic estimates (e.g. regression coefficient) AND variation (e.g. standard deviation) or associated estimates of uncertainty (e.g. confidence intervals)
☐	☒	For null hypothesis testing, the test statistic (e.g. *F*, *t*, *r*) with confidence intervals, effect sizes, degrees of freedom and *P* value noted<br>*Give P values as exact values whenever suitable.*
☒	☐	For Bayesian analysis, information on the choice of priors and Markov chain Monte Carlo settings
☒	☐	For hierarchical and complex designs, identification of the appropriate level for tests and full reporting of outcomes
☒	☐	Estimates of effect sizes (e.g. Cohen's *d*, Pearson's *r*), indicating how they were calculated

Our web collection on statistics for biologists contains articles on many of the points above.

Software and code

Policy information about availability of computer code

Data collection	No software was used.
Data analysis	PSI-BLAST v2.7.1 was used to detect protein homologs and MUSCLE v3.8 was used to align protein sequences. The python library scikit-learn v0.21.2 (https://scikit-learn.org/) was used to construct and run the random forest model. The custom code developed for this study is available on GitHub (https://github.com/gussow/acr).

For manuscripts utilizing custom algorithms or software that are central to the research but not yet described in published literature, software must be made available to editors and reviewers. We strongly encourage code deposition in a community repository (e.g. GitHub). See the Nature Research guidelines for submitting code & software for further information.

Data

Policy information about availability of data

All manuscripts must include a data availability statement. This statement should provide the following information, where applicable:

- Accession codes, unique identifiers, or web links for publicly available datasets
- A list of figures that have associated raw data
- A description of any restrictions on data availability

The authors declare that the data supporting the findings of this study are available within the paper and its supplementary information files. Supplementary Data 2 is available at ftp://ftp.ncbi.nih.gov/pub/wolf/_suppl/ACR20/supplementary_file_2.txt. The NCBI non-redundant sequence database is available at https://www.ncbi.nlm.nih.gov/.

Field-specific reporting

Please select the one below that is the best fit for your research. If you are not sure, read the appropriate sections before making your selection.

- [x] Life sciences
- [ ] Behavioural & social sciences
- [ ] Ecological, evolutionary & environmental sciences

For a reference copy of the document with all sections, see nature.com/documents/nr-reporting-summary-flat.pdf

Life sciences study design

All studies must disclose on these points even when the disclosure is negative.

Sample size	Sample sizes were determined based on the number of Acr families available.
Data exclusions	No data were excluded.
Replication	The model was retrained and tested on different portions of the data five times in 3-fold cross-validation to verify its robustness. The average AUC across the replications is 0.93 as stated in the manuscript, demonstrating that attempts at replication were successful and the model is robust.
Randomization	No controlled trial was conducted and therefore no randomization was required. As a supervised machine-learning study, the Acr status of each data point was used in the training algorithm to construct the model.
Blinding	No controlled trial was conducted, therefore no blinding was required to conduct this study. As a supervised machine-learning study, the Acr status of each data point was used in the training algorithm to construct the model.

Reporting for specific materials, systems and methods

We require information from authors about some types of materials, experimental systems and methods used in many studies. Here, indicate whether each material, system or method listed is relevant to your study. If you are not sure if a list item applies to your research, read the appropriate section before selecting a response.

Materials & experimental systems

n/a	Involved in the study	
x		Antibodies
x		Eukaryotic cell lines
x		Palaeontology and archaeology
x		Animals and other organisms
x		Human research participants
x		Clinical data
x		Dual use research of concern

Methods

n/a	Involved in the study	
x		ChIP-seq
x		Flow cytometry
x		MRI-based neuroimaging
